# Effects of first year COVID-19 pandemic on urology practice in three major Arab Countries: Sub-Analysis of a survey by Arab association of urology research group

**DOI:** 10.1371/journal.pone.0293458

**Published:** 2024-01-18

**Authors:** Basheer Elmohamady, Mohamed Omar, Amr S. El-Dakhakhny, Khalid Sayedahmed, Yahia Ghazwani, Saeed Bin Hamri, Abdullah Alkhayal, Khalid Alrabeeah, Wissam Kamal, Mohamed Abbasy, Yasser Farahat, Yasser A. Noureldin

**Affiliations:** 1 Department of Urology, Benha Faculty of Medicine, Benha University, Benha, Egypt; 2 Department of Urology, Menoufiya University Hospital, Menoufiya, Egypt; 3 College of Medicine, King Saud Bin Abdulaziz University for Health Sciences, Riyadh, KSA; 4 Division of Urology, King Abdulaziz Medical City, MNGHA, Riyadh, KSA; 5 Department of Urology, King Fahd Hospital, Jeddah, KSA; 6 Emergency Medicine Department, Northern Ontario School of Medicine, Thunder Bay, Ontario, Canada; 7 Department of Urology, Faculty of Medicine, Tanta University, Tanta, Egypt; 8 Urology Department, Sheikh Khalifa General Hospital, Umm Al Quwain, UAE; 9 Urology Department, Northern Ontario School of Medicine, Thunder Bay, Ontario, Canada; Alexandria University Faculty of Nursing, EGYPT

## Abstract

**Background and objectives:**

The World Health Organization (WHO) declared the coronavirus disease-19 (COVID-19) pandemic on March 11, 2020. The health care system faced tremendous challenges in providing ethical and high-quality care. The impact of COVID-19 on urological practices varied widely worldwide, including in Arab countries. This study aimed to compare the influence of the COVID-19 pandemic on urology practice in Egypt, the KSA, and the UAE during the first year of the pandemic.

**Methods:**

This sub-analysis assessed the demographics and COVID-19’s effects on urological practice in terms of adjustments to hospital policy, including outpatient consultations, the management of elective and urgent surgical cases, and the continuation of education across the three countries. The availability of personal protective equipment (PPE) and urologists’ emotional, physical, and verbal intimidation during COVID-19 were also compared.

**Results:**

Regarding the impact on hospital policy, consultations replaced by telemedicine were significantly higher in the KSA (36.15%), followed by the UAE (33.3%), then Egypt (10.4%) (P = 0.008). Elective cases requiring ICU admission were 65.1% in Egypt, 45.2% in the KSA, and 58.2% in the UAE and were performed only in high-risk patients. PPE was freely available in 20.8% of the Egyptian hospitals compared to 83.3% in the KSA and 81.8% in the UAE. Online courses were significantly higher in Egypt (70.8%), followed by the UAE (53%) and the KSA (41.7%) (P = 0.02). Emotional intimidation was higher than verbal intimidation, representing 80%, 75.9%, and 76% in the UAE, KSA, and Egypt, respectively.

**Conclusion:**

This sub-analysis outlined significant hospital policy changes across the three Arab countries. Exposure to emotional, verbal, and physical intimidation was observed. The development of teleconsultations and online platforms for educational purposes was observed.

## Introduction

At the beginning of January 2020, the 2019 novel coronavirus (2019-nCoV) was isolated in a Chinese city called Wuhan. The World Health Organization (WHO) mentioned it as Severe Acute Respiratory Syndrome Coronavirus 2 (SARS-CoV-2). The virus spread rapidly to more than 200 countries worldwide; as a result, WHO declared it a global pandemic on March 11, 2020. COVID-19 substantially impacted healthcare delivery and workers [1–4]. The insufficiency of personal protective equipment (PPE) exacerbated the condition in most hospitals worldwide. The workload of hospitals has increased considerably, and governments and hospital systems have taken many healthcare measures to adapt to this load. Many hospitals had to turn to quarantine to serve only COVID-19 patients [1, 2].

Residents of all specialties witnessed significant changes to their training environments [5]. Several national and international urology conferences offering hands-on training courses, including the European Association of Urology (EAU) and the American Urological Association (AUA) annual meetings, were postponed [6]. Therefore, innovative methods, such as online webinars, were created to keep physicians updated and their medical knowledge and spirit high during the challenging pandemic conditions [3]. To reduce exposure and maintain necessary PPE, the surgical volume was significantly reduced or even suspended [5]. Most surgical subspecialties canceled elective surgeries and served only emergency and non-deferrable oncologic surgeries. Social distancing became mandatory in the clinics, and the number of appointments was limited for urgent cases [1]. In some centers, clinics were prioritized, and non-urgent patients were consulted over Telemedia and postponed for at least six months. Moreover, case discussions and ward rounds were canceled [6].

Surgical training programs in vascular, general, and orthopedic surgery witnessed a dramatic decline, a delay in elective treatment, and a significant expansion of telemedicine services [7]. The rapid wave of COVID-19 cases has caused redistribution and reorganization of health services, including urology. Additionally, the decline in practical and surgical training hours affected urological trainees, who were not an exception [8].

To protect the patient’s and the surgeon’s safety, new methods have been devised to use minimally invasive surgeries for urological diseases during this pandemic. Additionally, hospitals had to comply with the varying degrees of elective operations that were less urgent and tightly restricted [9]. However, the basis of prioritization, deferral duration, the consequences of deferral and delay, and the success of alternative medical and surgical treatment methods were not evaluated [8].

Our previously published study [10] described significant changes to urological practice among urologists throughout the Arab world in the initial year of the COVID-19 pandemic. These alterations were most evident in hospitals’ regulations governing outpatient consultations, elective and emergency surgical procedures, and the transition to telemedicine. Arab urologists encountered significant difficulties in public and commercial sectors, with some experiencing verbal, emotional, and physical intimidation. While the transient changes in the Arab urological practice have been well documented [10], the differences among major Arab countries remain uncertain. Therefore, this sub-analysis was done to compare the influence of the COVID-19 pandemic during its initial year on urology practice in Egypt, the KSA, and the UAE.

## Materials and methods

### Study sampling and design

This quantitative, descriptive, comparative research was conducted through an online survey created and administered using a secure website utilizing Google Forms. The survey was distributed to every member of the Arab Association of Urology (AAU) through email in two waves, following approval from the AAU Board [10].

Our local ethics committee of Benha University revised this study and received approval (Rc-14-3-2023). Additionally, this study was conducted in line with the Declaration of Helsinki and its subsequent modifications. The voluntary survey completion was considered "written and verbal" consent to participate in the study. All ethical considerations were taken into account.

Both calls occurred in 2021, the first on January 7 and the second on February 15. During this time, two reminders were sent out. Respondents were urged to complete most questions since they were made obligatory. Before submitting their response, respondents could use the "Back" button to make any necessary changes to their responses. No respondent was offered the option to use the same email address to submit multiple responses.

The Arab Association of Urology (AAU) website was used to promote this poll, and the AAU offered a free one-year membership to the first ten respondents. The questionnaire was developed based on a review of relevant literature. Most of the questions were collected from a verified international survey conducted by the Société Internationale d’Urologie and published by Gravas et al. [11]. The authors included a few pertinent questions, such as intimidation exposure, the influence on private practice, and the psychological effects of COVID-19. The research team pre-tested the survey’s usability and technical issues to ensure that all the questions were appropriate.

The survey included a variety of open-ended, closed-ended, and Likert-scale questions to determine the participants’ age, gender, place of origin, kind of practice (private hospital, military hospital, academic hospital, or teaching hospital), and position (trainee, specialist, consultant, lecturer, assistant professor, or professor).

The survey evaluated the effects of the COVID-19 pandemic on various facets of urological practice, including changes to hospital policies for elective surgical cases, particularly those necessitating intensive care unit admission. It also assessed changes in the outpatient clinic activities, including complete closure, replacement by telemedicine, restriction to follow-ups only, and limitation to specific cases. Moreover, the triage policy was evaluated for some common urological procedures, such as radical cystectomy, varicocelectomy, transurethral resection of the prostate, transurethral resection of bladder tumor, radical nephrectomy, partial nephrectomy, non-obstructive stones, obstructive stones, and radical nephrectomy, on a scale of 0 to 5, with 0 denoting the lowest priority and 5 the highest.

The survey included the shift in hospital policies for emergency cases, such as those requiring immediate intervention, and the policies related to surgical tools, including the use of disposable scopes. Furthermore, the survey covered other topics such as the availability and type of PPE, ongoing medical education, private practice, psychological and behavioural health, and intimidating exposure.

There was no collection of any personally identifiable data. To avoid unwanted access, anonymized data were electronically collected in Google Forms before being transferred and saved in an electronic spreadsheet format (Microsoft Excel 2010, Microsoft Corporation, Redmond, WA).

### Statistical methods

The sample size for the original survey was calculated using Epi-info software version 7.2.5.0 based on an expected delay in the urological services of 31% according to Teoh et al. The minimum sample size calculated was 128 participants. The margin of error and confidence level were adjusted at 8% and 95%, respectively [2]. SPSS Version 26 was used for data analysis (IBM Corp., Armonk, NY, USA). Descriptive statistics were done using numbers and percentages. All findings were compared between the three Arab countries using the Chi-square or Fisher’s exact test. All statistical tests were two-sided. Significance was defined as a P-value less than 0.05.

## Results

Two hundred fifty-five AAU members from three Arab nations (Egypt, the KSA, and the UAE) responded to this survey. The majority were males. Participants’ demographics are presented in Table 1. The position was significantly different among the participants (P = 0.001). Most were consultants and specialists from the UAE (34.8% and 43.9%, respectively). Academic hospitals were significantly higher in Egypt (P < 0.001). Teaching and military hospitals were significantly higher in the KSA (P = 0.004 and 0.002, respectively), while private hospitals were significantly higher in the UAE (P = 0.001) (**Table 1).**

**Table 1:** Demographics of participants.

Variables (255 respondents)		Arab Republic of Egypt (48)		Kingdom of Saudi Arabia (36)		United Arab Emirates (66)		p-value
		Number	(%)	Number	(%)	Number	(%)	
**Age (years)**	<40	22	45.8%	9	25.0%	9	13.6%	0.01
	40–50	14	29.2%	17	47.2%	26	39.4%	
	51–60	5	10.4%	6	16.7%	18	27.3%	
	61–65	5	10.4%	3	8.3%	6	9.1%	
	>65	2	4.2%	1	2.8%	7	10.6%	
**Gender**	Males	48	100.0%	36	100.0%	61	92.4%	0.05
	Females	0	0.0%	0	0.0%	5	7.6%	
**Type of practice**	Academic hospital	30	62.5%	7	19.4%	13	19.7%	<0.001
	Teaching hospital	5	10.4%	15	41.7%	16	24.2%	0.004
	Private hospital	14	29.2%	14	38.9%	42	63.6%	0.001
	Military hospital	2	4.2%	5	13.9%	0	0.0%	0.002
	Insurance hospital	7	14.6%	3	8.3%	2	3.0%	0.08
**Position**	Professor	9	18.8%	4	11.1%	4	6.1%	0.001
	Assistant professor	10	20.8%	1	2.8%	2	3.0%	
	Lecturer	1	2.1%	0	0.0%	1	1.5%	
	Consultant	6	12.5%	15	41.7%	23	34.8%	
	Specialist	16	33.3%	15	41.7%	29	43.9%	
	Trainee	6	12.5%	1	2.8%	7	10.6%	

Regarding the impact on hospital policy, telemedicine was significantly higher in the KSA (36.15%), followed by the UAE (33.3%) and Egypt (10.4%) (P = 0.008). Completely closed and restricted consultations for follow-up and specific cases were insignificantly different between the studied countries (P = 0.2, 0.09, and 0.4, respectively) (**Table 2).**

**Table 2:** Effects on the hospital policy.

Variable		Arab Republic of Egypt		Kingdom of Saudi Arabia		United Arab Emirates		p-value
**Consultations at outpatient clinic**	Completely closed	12	25.0%	7	19.4%	8	12.1%	0.2
	Replaced by telemedicine	5	10.4%	13	36.1%	22	33.3%	0.008
	Restricted for follow-up only	4	8.3%	0	0.0%	7	10.6%	0.09
	Restricted for specific cases only	16	33.3%	10	27.8%	26	39.4%	0.4
	No change, fully work	17	35.4%	9	25.0%	18	27.3%	0.5
**Policy for elective operative cases** *	Elective surgery reduced by >25%	10	23.3%	2	6.5%	4	7.3%	0.144
	Elective surgery reduced by >25% <50%	12	27.9%	10	32.3%	13	23.6%	
	Elective surgery reduced by >50% -<75%	8	18.6%	7	22.6%	8	14.5%	
	Elective surgery reduced by >75%	9	20.9%	8	25.8%	16	29.1%	
	No elective surgery right now	4	9.3%	4	12.9%	14	25.5%	
**Policy for elective cases require ICU admission** *	Performed as in the past	1	2.3%	0	0.0%	3	5.5%	0.24
	Performed if high risk of disease progression	28	65.1%	14	45.2%	32	58.2%	
	Postponed	14	32.6%	17	54.8%	20	36.4%	
**Policy regarding preoperative COVID-19 testing**	Case by case based on a committee decision	6	12.5%	4	11.1%	0	0.0%	NA
	Risky patients with bad general conditions	1	2.1%	0	0.0%	0	0.0%	
	Patients suspicious of Covid-19 infection	25	52.1%	10	27.8%	1	1.5%	
	All patients	11	22.9%	22	61.1%	64	97.0%	
	None	5	10.4%	0	0.0%	1	1.5%	
**Policy regarding the use of PPE while in the hospital**	Surgical mask	39	81.3%	27	75.0%	40	60.6%	0.55
	Goggles	2	4.2%	8	22.2%	22	33.3%	0.06
	Face shield	20	41.7%	12	33.3%	30	45.5%	0.12
	N95 or FFP3 mask	12	25.0%	19	52.8%	39	59.1%	0.09

PPE: personal protective equipment, P< 0.05 was considered significant

* percentages were calculated based on total participants with policy changes in their hospitals regarding elective cases; NA: Not applicable.

Respondents in the three countries reported changes to hospital policies regarding elective urology interventions without a significant difference among the studied countries (P = 0.144). Approximately one quarter (25.5%) reported cancellation of elective interventions in the UAE, compared to 12.9% in the KSA and 9.4% in Egypt (**Table 2).**

**Fig 1:**
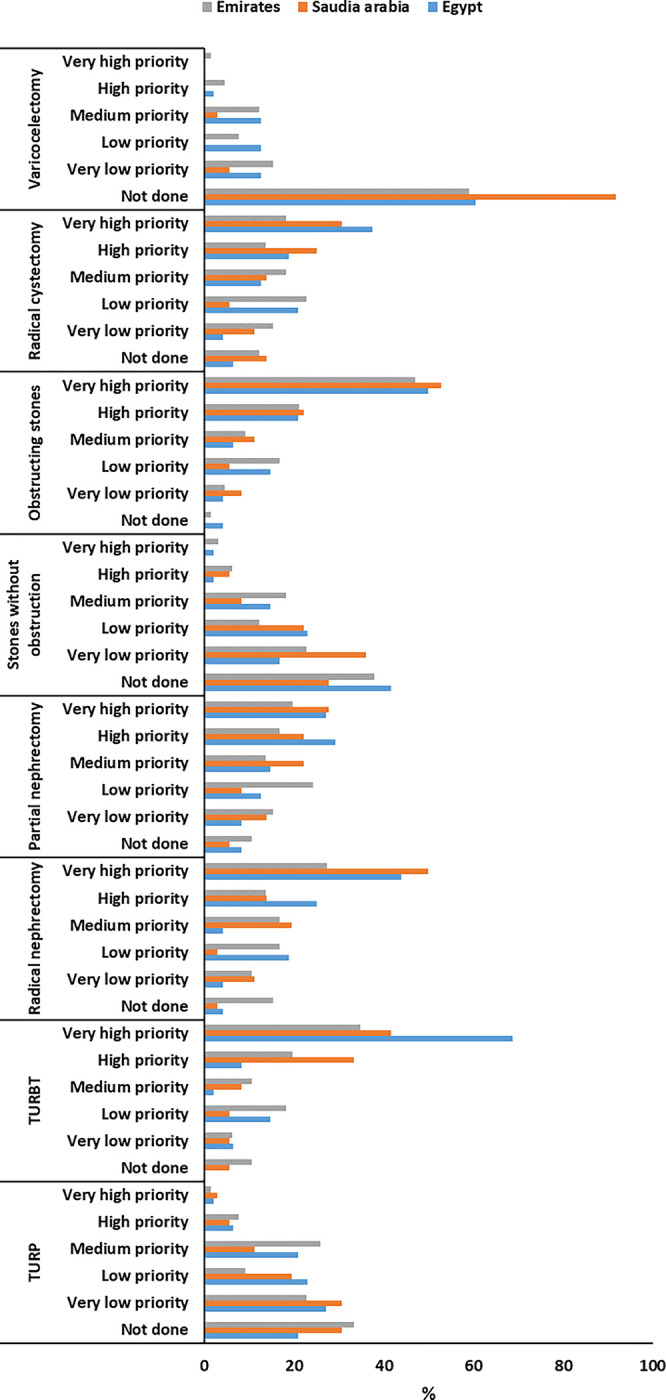
Impact of the first year of COVID-19 on eight frequently performed urology surgeries.

Regarding policies for elective cases needing ICU admission, no significant difference was reported between the studied countries (P = 0.240). Surgeries, such as TURBT and radical nephrectomy, were highly prioritized compared to the other procedures in the studied countries. Varicocelectomy and stones without obstruction had the lowest priority and were not done in most cases (**Table 2 and Fig 1).**

In the UAE, most respondents (97%) reported that preoperative COVID-19 testing was available to all patients, compared to 61.1% in the KSA and 22.9% in Egypt. On the other hand, 52.1% of Egyptian respondents reported preoperative COVID-19 testing was only offered for patients suspected of COVID-19, compared to 27.8% in the KSA and 1.5% in the UAE (**Table 2).**

No significant difference was detected in hospital PPE use, including surgical masks, goggles, face shields, and N95 or FFP3 masks (P = 0.55, 0.06, 0.12, and 0.09, respectively) (**Table 2).**

PPE availability significantly differed between the studied countries (P < 0.001). About one-quarter (20.8%) reported that PPE was readily available in Egyptian hospitals compared to 83.3% and 81.8% in the KSA and UAE, respectively. In contrast, 46.6% reported limited availability in Egyptian hospitals compared to 16.7% and 13.6% in the KSA and UAE, respectively. Only 14.6% reported no availability in the Egyptian hospitals, compared to 0.0% and 4.5% in the KSA and the UAE, respectively (**Table 3).**

**Table 3:** Effects on the urologists.

Variable		Arab Republic of Egypt		Kingdom of Saudi Arabia		United Arab Emirates		p-value
**Was PPE provided by the hospital?**	No, we had to buy it ourselves	7	14.6%	0	0.0%	3	4.5%	<0.001
	Yes, but with very limited availability	31	64.6%	6	16.7%	9	13.6%	
	Yes, the hospital provides all types of PPE	10	20.8%	30	83.3%	54	81.8%	
**Continuing education during COVID-19**	Online webinars	43	89.6%	36	100.0%	64	97.0%	0.06
	Online courses	34	70.8%	15	41.7%	35	53.0%	0.02
	Online videos	30	62.5%	17	47.2%	35	53.0%	0.30
**Private practice during COVID-19** *	No change	3	8.6%	2	20.0%	4	15.4%	0.34
	Only emergency	1	2.9%	1	10.0%	1	3.8%	
	Severe decrease in patients’ number	31	88.6%	7	70.0%	19	73.1%	
	Slight decrease in patients’ number	0	0.0%	0	0.0%	2	7.7%	
**Intimidation during COVID-19** **	Emotional	19	76.0%	10	76.9%	12	80.0%	0.62
	Physical	0	0.0%	1	7.7%	1	6.7%	
	Verbal	6	24.0%	2	15.4%	2	13.3%	

PPE: personal protective equipment

*percentages were calculated based on those who had private practice

** percentages were calculated based on those who were subjected to intimidation.

Most respondents stated that during COVID-19, urology education changed, forcing them to use online learning methods. Continuing education with online courses was significantly different among the studied countries. The highest was observed in Egypt (70.8%), followed by the UAE (53.0%) and the KSA (41.7%) (P = 0.02). Online webinars and videos were insignificantly different between the studied countries (P = 0.06 and 0.30, respectively) (**Table 3).**

Regarding private practice during COVID-19, there was a severe decline in the number of patients, with an insignificant difference between the studied countries (P = 0.340) (**Table 3).**

**Fig 2:**
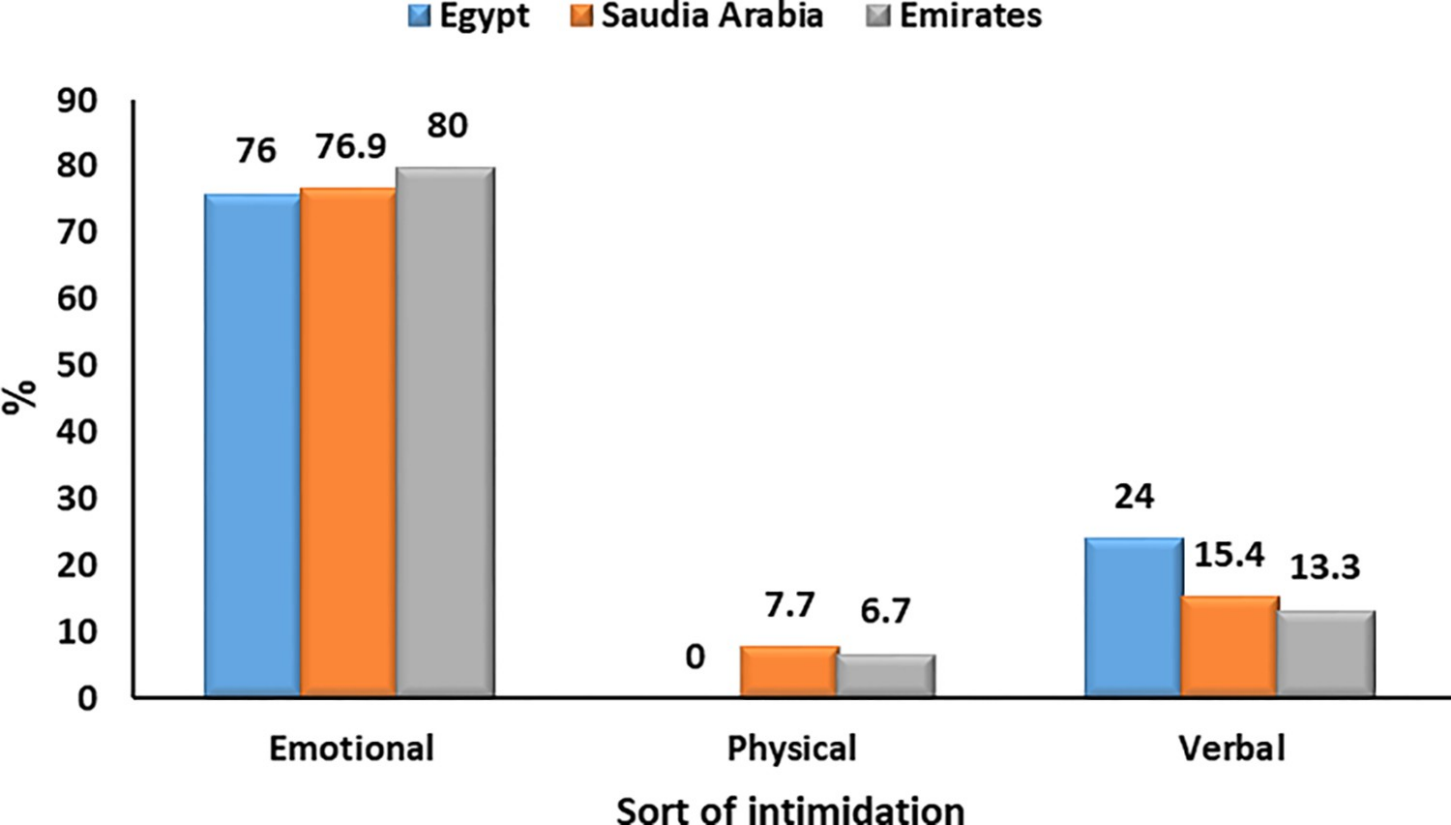
Intimidations to urologists during the first year of COVID-19 in Egypt, Saudi Arabia, and the Emirates.

Respondents were subjected to emotional, physical, and verbal intimidation. Emotional intimidation was the highest (80% in the UAE, 75.9% in the KSA, and 76% in Egypt), with no significant difference between the studied countries (P = 0.62) (**Table 3 and Fig 2).**

## Discussion

The COVID-19 pandemic is considered the worst challenge to healthcare systems worldwide, resulting in a shift to a community-centered approach instead of patient-centered medical care [6].

The EAU has divided elective urological interventions based on the timeline of clinical harm into low risk (no harm by 6 months), intermediate risk (no harm by 3–4 months), and high risk (no harm by 6 weeks). Therefore, selecting the modality of surgical intervention was a crucial parameter during the COVID pandemic and was based on the available scientific and social evidence [6, 12].

The Urology Collaborative Online Video Didactics, utilizing the internet during the COVID-19 pandemic, offered a lecture series on Zoom. The New York section of the AUA also offered the Educational Multi-institutional Program for Instructing Residents (EMPIRE). Many of these Telemedia lectures were recorded as resources for urology education and activities [2].

All health systems worldwide have struggled to deal with the COVID-19 epidemic due to the unexpected surge in cases [13]. The effect of rescheduling and postponing the procedures may have harmed patients, especially when the time frame was not even predictable. Therefore, the urological care priority and selecting the intervention modality during that pandemic should have been based on scientific evidence and local strategic context [1]. Different demands have necessitated reconfiguring the usual healthcare service delivery model worldwide, including Arab countries, which are the main focus of the current study [13, 14].

In the present study, telemedicine use in urology was significantly higher in the KSA (36.15%), followed by the UAE (33.3%) and Egypt (10.4%). This positive attitude towards telemedicine in the KSA was similarly reported by Alajwari et al. [15]. Most participants (70.0%) believed telemedicine might lower transportation expenses and save time, labour, and costs. In Dubai, Al-Sharif et al. [16] reported high satisfaction levels with telemedicine but identified specific areas for improvement. They found that patients with higher education and those who experienced a shorter duration of teleconsultations were more satisfied, so it is recommended that guidelines define the optimal telemedicine consultation duration.

From an Egyptian perspective, Abdelmotagly et al. [17] evaluated 200 patients’ experiences with remote urology clinics and concluded that half of the patients (46%) prefer face-to-face consultations. This result could be explained by the need for physical examination (e.g., inguinoscrotal and penile referrals) or a language or hearing barrier. Therefore, it is essential to triage patients and provide them with thorough information about what to expect from remote consultation [18].

In our study, more than one-third of elective surgeries were postponed in Egypt and the UAE, compared to half in the KSA. This finding is consistent with the recommendations of international organizations and the measures adopted to mitigate the negative impacts of the COVID-19 epidemic [19–21]. Most Saudi governmental hospitals applied these measures [22].

Egypt recorded the highest rates (65.1%), followed by the UAE (58.2%) and the KSA (45.2%) in terms of high-priority surgeries such as kidney stone obstruction and cancer operations, including TURBT and radical nephrectomy. In line with these findings, Raheem et al. [23] indicated that urology elective treatments in the KSA declined by 34.3% in the first three months of 2020 compared to the same period in 2019, along with prioritizing emergency interventions to prevent permanent disease development or organ destruction, such as urolithiasis-related procedures.

The use of PPE was essential in urology because most patients are elderly and have a variety of co-morbidities, rendering them highly susceptible to COVID-19 [24]. Due to the disruption in global medical supply networks, there was a fear of running out of supplies worldwide [25]. Based on our findings, only 20% reported that Egyptian hospitals had free access to PPE, which is available in most Saudi and Emirati hospitals. This shortage could be explained by the fact that the Egyptian market heavily depends on imported medical equipment. Imports were halted during the initial COVID-19 wave, resulting in severe shortages [26]. Furthermore, Osman et al. [27] revealed that less than one-third of Egyptian healthcare workers wore throwaway clothing or scrubs due to the lack of PPE. Consequently, local infection control teams recommended various techniques to reuse face masks and respirators [26].

Urological education had to change quickly in response to the COVID-19 epidemic [28, 29]. Our observations revealed that 70.8% of urologists in Egypt continued their education online, compared to 53% and 147% in the UAE and KSA, respectively. Omil-Lima et al. [30] proved the effectiveness of an online urology course. They believed that online education would continue after the COVID-19 pandemic. Additionally, Tanidir Y et al. [3] proved that the webinar was an effective method for sharing scientific information with many advantages, including low cost and reaching many participants virtually without contact. Although COVID-19 vaccines have been developed, there is a common perspective that the pandemic will last beyond 2022 [31]. As a result, virtual learning continues to be a vital tool for medical education across the entire field of urology.

Globally, there was relevant mental stress and uncertainty about the COVID-19 pandemic among urologists [32–34]. The psychological discomfort and the intense fear of infection were significant burdens [29]. Residents were frequently kept apart from their loved ones to curb the spread of COVID-19, resulting in heightened isolation [35]. The adverse emotional effects of the pandemic have been observed in the healthcare field, particularly concerning the long working hours and social distancing [3].

According to the current study, urologists suffered from intimidation for being doctors, whether emotional, physical, or verbal, with insignificant differences among the three countries. However, emotional intimidation was the most prevalent in the UAE, Egypt, and the KSA (80%, 76%, and 75.9%, respectively). These alarming findings demonstrate how supporting and monitoring urologists’ mental well-being during epidemics is just as crucial as maintaining their physical health [28, 32].

This study has some limitations. The study was cross-sectional, and the overall response rate was not reported. Additionally, recall bias might have affected the results. Nevertheless, this is the first study to compare three major Arab countries regarding urology practice from various perspectives during the COVID-19 pandemic.

## Conclusion

The COVID-19 pandemic has imposed many challenges on urology healthcare workers. A substantial proportion of urologists reported insufficient training and the lack of PPE during the 1st year of the pandemic in the three countries. While telemedicine was successfully implemented in managing Saudi and Emirati patients, further enhancements are needed in Egypt. The flexibility of the three countries in managing elective cases contributed to reducing the spread of COVID-19. In contrast to KSA and UAE, Egypt reported inadequate PPE availability. Urologists across the studied countries responded well to online education to fill the gap during the first year of the COVID-19 pandemic. Finally, different aspects of intimidation were reported, which might have added to the load on healthcare workers in the three Arab countries. We believe that the results of the present study will help the urology departments in these three countries take the required precautions for any possible future mass casualty events.
